# Supplementary material for: Resilience, Perceived Stress from Adapted Medical Education Related to Depression among Medical Students during the COVID-19 Pandemic
Source: Healthcare (Basel). 2023 Jan 12;11(2):237. doi: 10.3390/healthcare11020237 (PMC9859097; doi:10.3390/healthcare11020237)
Supplement: Supplementary file 1 [file healthcare-11-00237-s001.zip › Material S2.pdf]

Perceived stress of AMEC				
**Instruction:** Please rate to what extent the following events causes you stress	**level of stress**			
**Loss opportunity for clinical practices and experiments,**	No impact	Mild	Moderate	Severe
**Lack of peer contact**	No impact	Mild	Moderate	Severe
**Lack of face-to-face communication with teachers**	No impact	Mild	Moderate	Severe
**Online examination**	No impact	Mild	Moderate	Severe
