# Supplementary material for: Resilience, Perceived Stress from Adapted Medical Education Related to Depression among Medical Students during the COVID-19 Pandemic
Source: Healthcare (Basel). 2023 Jan 12;11(2):237. doi: 10.3390/healthcare11020237 (PMC9859097; doi:10.3390/healthcare11020237)
Supplement: Supplementary file 1 [file healthcare-11-00237-s001.zip › Material S3.pdf]

RI-9

Instruction: Consider the following statements, how well they describe your thoughts and expressions by marking ✓ in the box that best matches your thoughts and expressions

1 = does not describe me at all
2 = does not quite describe me
3 = describe me somewhat
4 = describe me quite well
5 = describe me very well

Feelings and expressions that describe you	1	2	3	4	5
1. I believe that I must overcome the obstacles I face.	☐	☐	☐	☐	☐
2 I can withstand the pressure.	☐	☐	☐	☐	☐
3 I am proud that I can overcome all the bad things in life.	☐	☐	☐	☐	☐
4 I believe I have the ability to overcome obstacles.	☐	☐	☐	☐	☐
5 When faced with a problem, it made me active to fight.	☐	☐	☐	☐	☐
6 No matter what happens, I believe that I am always fully aware of it.	☐	☐	☐	☐	☐
7 I think I can overcome the problem because I am a talented person.	☐	☐	☐	☐	☐
8 I considered the period when the problem caused me to learn.	☐	☐	☐	☐	☐
9 I believe that in times of crisis there is always an opportunity.	☐	☐	☐	☐	☐

Copyrighted 2020

Resilience Inventory-9 (RI-9): Prof. Tinakon Wongpakaran
& Prof. Nahathai Wongpakaran, Psychotherapy Unit and Geriatric Unit,
Department of Psychiatry, Faculty of Medicine, Chiang Mai University, Thailand
